# Mapping Total Exceedance PM_2.5_ Exposure Risk by Coupling Social Media Data and Population Modeling Data

**DOI:** 10.1029/2021GH000468

**Published:** 2021-11-01

**Authors:** Zheng Cao, Guanhua Guo, Zhifeng Wu, Shaoying Li, Hui Sun, Wenchuan Guan

**Affiliations:** ^1^ School of Geographical Sciences Guangzhou University Guangzhou China; ^2^ Southern Marine Science and Engineering Guangdong Laboratory (Guangzhou) Guangzhou China

**Keywords:** exceedance PM_2.5_ exposure risk, total people groups, social media data, population modeling data, risk assessment

## Abstract

The PM_2.5_ exposure risk assessment is the foundation to reduce its adverse effects. Population survey‐related data have been deficient in high spatiotemporal detailed descriptions. Social media data can quantify the PM_2.5_ exposure risk at high spatiotemporal resolutions. However, due to the no‐sample characteristics of social media data, PM_2.5_ exposure risk for older adults is absent. We proposed combining social media data and population survey‐derived data to map the total PM_2.5_ exposure risk. Hourly exceedance PM_2.5_ exposure risk indicators based on population modeling (HEPE_pmd_) and social media data (HEPE_sm_) were developed. Daily accumulative HEPE_sm_ and HEPE_psd_ ranged from 0 to 0.009 and 0 to 0.026, respectively. Three peaks of HEPE_sm_ and HEPE_psd_ were observed at 13:00, 18:00, and 22:00. The peak value of HEPE_sm_ increased with time, which exhibited a reverse trend to HEPE_psd_. The spatial center of HEPE_sm_ moved from the northwest of the study area to the center. The spatial center of HEPE_psd_ moved from the northwest of the study area to the southwest of the study area. The expansion area of HEPE_sm_ was nearly 1.5 times larger than that of HEPE_psd_. The expansion areas of HEPE_psd_ aggregated in the old downtown, in which the contribution of HEPE_psd_ was greater than 90%. Thus, this study introduced various source data to build an easier and reliable method to map total exceedance PM_2.5_ exposure risk. Consequently, exposure risk results provided foundations to develop PM_2.5_ pollution mitigation strategies as well as scientific supports for sustainability and eco‐health achievement.

## Key Points

- Total exceedance PM_2.5_ exposure risk, including youths and older adults, was mapped
- The hourly exceedance PM_2.5_ exposure risk (HEPE)_psd_ was more aggregated than the HEPE_sm_
- Contribution of HEPE_psd_ varied geographically with percentage more than 50%

## 1 Introduction

The adverse effects of PM_2.5_ on public health have become a worldwide concern since the last century (Cohen et al., 2017; Dockery et al., 1993; Kioumourtzoglou et al., 2015; Lelieveld et al., 2015; Pope et al., 2002). As one of the largest developing countries, a nearly twofold increase in the population‐weighted PM_2.5_ exposure risk has been observed in China since 1990 (Brauer et al., 2015; Y. Chen et al., 2013; Huang et al., 2014). The projection of PM_2.5_ suggests that under all emission scenarios, the PM_2.5_ concentration continues to increase, which implies that air pollution is a crucial threat to public health (Apte et al., 2015; Jie et al., 2015; Qin et al., 2015). These findings provide vital information on the estimation of PM_2.5_ exposure risk to public health.

In previous studies, daily mean PM_2.5_ concentration and population survey data were applied to construct indicators to calculate the population‐weighted PM_2.5_ exposure risk (Franklin et al., 2008; Pascal et al., 2014; Vodonos et al., 2018; Y. Wang et al., 2017). There are two disadvantages to this method. First, the daily mean PM_2.5_ concentration value cannot fully illustrate the hourly variations in PM_2.5_. Moreover, the hourly PM_2.5_ concentration value might be higher than the daily mean PM_2.5_ concentration as well as the PM_2.5_ health guideline threshold set by the World Health Organization, which leads to an underestimation of its adverse effects on public health (Lin et al., 2017, 2018). Second, this method is confined to the low spatiotemporal resolution of the population survey data. The precise details of the PM_2.5_ exposure risk are not well described. Therefore, more scientific indicators are necessary to improve the assessment results of the PM_2.5_ exposure risk.

Therefore, social media data have been introduced into related investigations, which refer to the online population footprints collected by smartphones and facilities. With the prevalence of social media, these data are widely applied in population mobility‐related investigations, such as urban function zone extractions, urban expansion, and population commuting (Li, Lyu, Huang, et al., 2020; Li, Lyu, Liu et al., 2020; Shelton et al., 2015; Shen & Karimi, 2016; Q. Wang et al., 2018; Ye et al., 2020). Combined with the definition of the exceedance PM_2.5_ exposure risk and social media data, a novel indicator called hourly exceedance PM_2.5_ exposure risk (HEPE) was constructed. A high spatiotemporal resolution of population‐weighted PM_2.5_ exposure risk variations can be obtained (Cao et al., 2020, 2021). However, social media data are regarded as nonrepresentative and non‐sample data. Social media data are collected from smartphones, which are used less often by older adults. This situation results in uncertainties in the population‐weighted PM_2.5_ exposure risk assessment (Song et al., 2019; Yuan et al., 2020). Therefore, the population‐weighted PM_2.5_ exposure risk, relying only on social media data or population survey data, cannot fully reflect the total risk. A quantitative assessment of the total population‐weighted PM_2.5_ exposure risk at a high spatiotemporal resolution remains unsolved.

Therefore, we plan to assess the total PM_2.5_ exposure risk by combining population survey‐related data and social media data using the indicator designed in our latest investigation named the HEPE (Cao et al., 2020). First, we constructed the HEPE using population survey‐related data and social media data. Then, the spatiotemporal variations in the HEPE considering different data sources were quantified. Finally, the contribution of the HEPE considering the population survey‐related data to the total HEPE was evaluated. The findings from this study provide new insights that can be combined with different data sources to conduct public health‐related investigations. By considering different data sources, maps of air pollution for different population groups were obtained. This is a vital foundation for air pollution mitigation strategies.

## 2 Study Area

**Figure 1:**
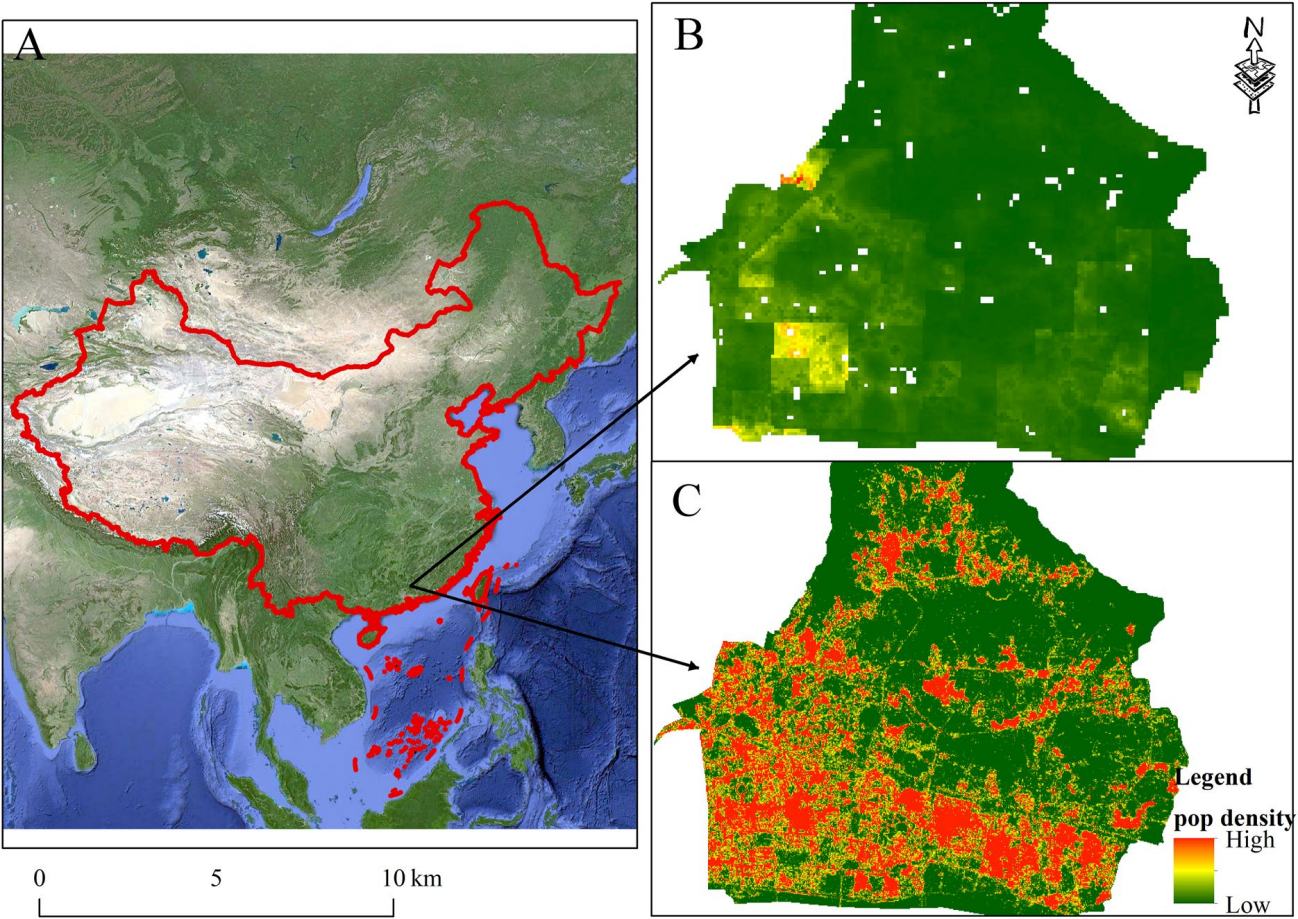
Location of the study area and spatial pattern of population obtained from different data source. Panel (a) shows the location of study area, panel (b) shows a spatial distribution of population over 60 years old in the study area obtained from modeling data, and panel (c) shows a spatial distribution of population in the study area obtained from social media data.

The Tianhe District is the economic center of Guangzhou, located between 23.24°N–23.04°N and 113.18°E–113.45°E (Figure 1). Economic development has been clearly observed since 1979. In Guangzhou, the proportion of the gross domestic product in Tianhe increased from less than 10.00% in 1976 to 21.36% in 2020. The significant economic development has generated great demands for energy consumption and individual wealth, which has resulted in an increase in air pollution, such as PM_2.5_. As a central downtown area, mostly indigenous people and immigrants inhabit this area. Most of them were older adults with low education levels. Thus, an awareness of air pollution protection is absent. Therefore, conducting air pollution assessments in this area is essential and urgent.

## 3 Data and Methodology

### 3.1 Data

#### 3.1.1 Population Survey‐Related Data

The global age‐group composition data for 2018 were obtained from WorldPop (https://www.worldpop.org/). The study population was divided into 36 groups, separated by gender, in the age groups of less than 1 year old, 1–20 years old, 20–25 years old, 25–30 years old, 30–35 years old, 35–40 years old, 40–45 years old, 45–50 years old, 50–55 years old, 55–60 years old, 60–65 years old, 65–70 years old, 70–75 years old, 75–80 years old, and older than 80 years old. The spatial resolution was 100 m. To map the accurate and precise population age group data sets, the random forest method was used considering the population census database, land cover, human settlement, and other 300 input data (Stevens et al., 2015; Tatem et al., 2013). For the sake of accuracy and high spatial resolution, these data are widely used in population‐based investigations, such as infectious transmission risk assessment, population distribution mapping validations, and specific population group location mapping (Giles et al., 2020; Lai et al., 2020; Leasure et al., 2020; Lloyd et al., 2020; Ruktanonchai et al., 2020). Furthermore, individuals older than 60 years old, female and male, were included in this investigation as the representatives of the older adult group.

#### 3.1.2 Social Media Data

Tencent user density data, which were collected from the Tencent platform, were used as the social media data in this study. These data were generated when smartphone users activated Tencent applications, such as WeChat (a social chatting application), Tencent QQ (an immediate message application), and Tencent map (a navigation application). Tencent applications cover more than 93% of smartphone users in Guangzhou. The spatiotemporal resolution of this data was 25 m and 1 hr. The prevalence of this data has been widely applied in population clustering pattern recognition analysis (Y. Chen et al., 2019; Niu et al., 2020), urban transportation analysis (Li, Lyu, Huang, et al., 2020; Li, Lyu, Liu et al., 2020), and public health risk assessments (Cao et al., 2020; Zheng et al., 2020).

#### 3.1.3 Air Pollution Monitoring Data

Hourly PM_2.5_ monitoring data were obtained from the Guangdong Environmental Monitoring Platform (http://gdee.gd.gov.cn). The hourly mean PM_2.5_ concentration was collected from 11 stations for May 17, 2019. Data quality was controlled, and the invalid and missing data were deleted.

### 3.2 Methodology

#### 3.2.1 HEPE Indicator Development

The HEPE developed in our previous investigation was applied in this study (Zheng et al., 2020). This indicator was constructed considering the exceedance PM_2.5_. Two HEPE indicators were calculated based on the Tencent user density data (TUD) and population survey‐related data. The calculations are as follows:

(1) $${\text{HEPE}}_{\text{sm}}=\frac{{\text{NTUD}}_{i}}{\sum_{n}^{i=1}{\text{NTUD}}_{i}}\times 100\%\times {\text{EP}}_{i}$$

(2) $${\text{HEPE}}_{\text{psd}}=\frac{{\text{NPSD}}_{i}}{\sum_{n}^{i=1}{\text{NPSD}}_{i}}\times 100\%\times {\text{EP}}_{i},$$

where HEPE_sm_ refers to the HEPE calculation results based on the TUD data, HEPE_pmd_ refers to the HEPE calculation results based on the population modeling data, and EP_*i*_ refers to the exceedance PM_2.5_ concentration beyond 25 µg/m^3^ at grid *i*. Also, 25 µg/m^3^ has been set as the health safety level guided by the World Health Organization. NTUD_*i*_ and NPSD_*i*_ were the normalized results of the Tencent user density data and population modeling data, respectively, for the sake of different dimensions.

#### 3.2.2 Spatiotemporal Pattern Detecting of HEPE

Spatiotemporal variations were detected using the standard deviation ellipse method (SDEM). Three dimensions are considered in the SDEM: major extension direction, secondary extension direction, and spatial center. Long and short diameters represent the major extension and secondary extension directions, respectively. The location information of the spatial center denotes the distribution center of the HEPE. The calculation is as follows:

(3) $$M\left(\bar{X},\bar{Y}\right)=\left(\frac{\sum_{i=1}^{n}w_{i}x_{i}}{\sum_{i=1}^{n}w_{i}},\frac{\sum_{i=1}^{n}w_{i}y_{i}}{\sum_{i=1}^{n}w_{i}}\right)$$

(4) $$\tan\;\theta =\frac{\left[(\sum_{i=1}^{n}w_{i}^{2}x_{i}^{2}-\sum_{i=1}^{n}w_{i}^{2}y_{i}^{2})+\sqrt{{\left(\sum_{i=1}^{n}w_{i}^{2}x_{i}^{2}-\sum_{i=1}^{n}w_{i}^{2}y_{i}^{2}\right)}^{2}+4\left(\sum_{i=1}^{n}w_{i}^{2}x_{i}^{2}y_{i}^{2}\right)}\right]}{2\sum_{i=1}^{n}w_{i}^{2}x_{i}^{\prime }y_{i}^{\prime }}$$

(5) $$\sigma_{x}=\frac{\sqrt{\sum_{i=1}^{n}{\left(w_{i}x_{i}^{\prime }\;\cos\;\theta -w_{i}y_{i}^{\prime }\;\sin\;\theta \right)}^{2}}}{\sum_{i=1}^{n}w_{i}^{2}}$$

(6) $$\sigma_{y}=\frac{\sqrt{\sum_{i=1}^{n}{\left(w_{i}x_{i}^{\prime }\;\sin\;\theta -w_{i}y_{i}^{\prime }\;\cos\;\theta \right)}^{2}}}{\sum_{i=1}^{n}w_{i}^{2}}$$

where $M\left(\bar{X},\bar{Y}\right)$ represents the coordinate information of spatial center, *θ* represents the angle between the long diameter and north direction, *x*_*i*_ and *y*_*i*_ represent the coordinate information of grid *i*, $x_{i}^{\prime }$ and $y_{i}^{\prime }$ represent the standard deviation of the spatial distance standard between grid *i* and the spatial center, and *σ*_*x*_ and *σ*_*y*_ represent the standard deviation along the *X* axis and *Y* axis, respectively.

## 4 Results

### 4.1 Dynamics of Exceedance PM_2.5_ Exposure Risk Considering Social Media Data and Population Modeling Data

**Figure 2:**
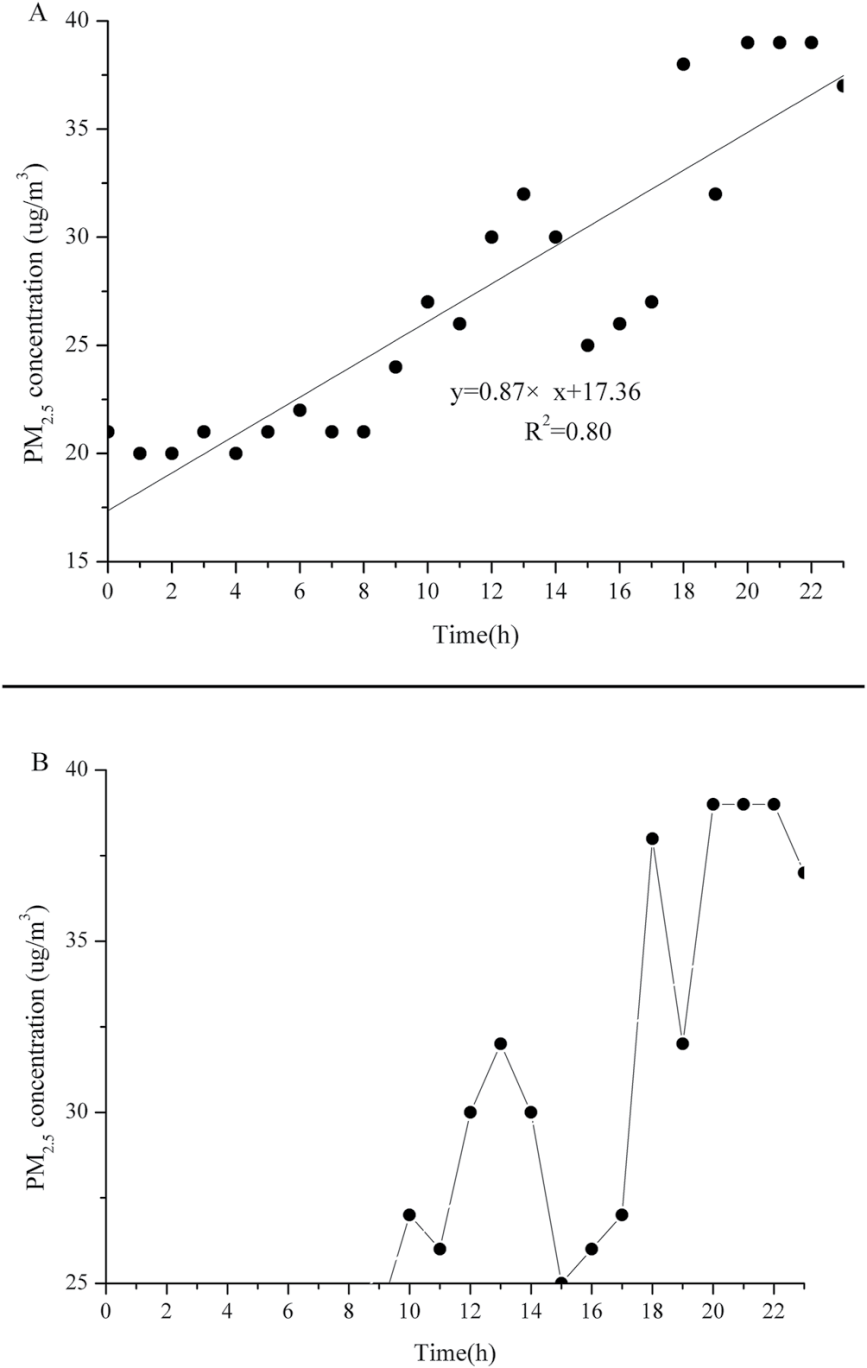
Temporal variation characteristics of monitored PM_2.5_ concentration. Panel (a) shows the temporal trend of PM_2.5_ concentration and panel (b) shows the temporal variation of exceedance PM_2.5_ concentration.

As Figure 2 illustrated, the linear regression model showed increasing trends of temporal variation characteristics of PM_2.5_ concentration at hourly level. The temporal trend slope was 0.87, which indicated a 0.87 µg/m^3^ increasing per hour. The first exceedance PM_2.5_ concentration value was observed at 10:00. The lowest exceedance PM_2.5_ concentration was found at 17:00 with a value of 0.25 µg/m^3^. The highest exceedance PM_2.5_ concentration was found at 22:00 with a value of 14.09 µg/m^3^.

**Figure 3:**
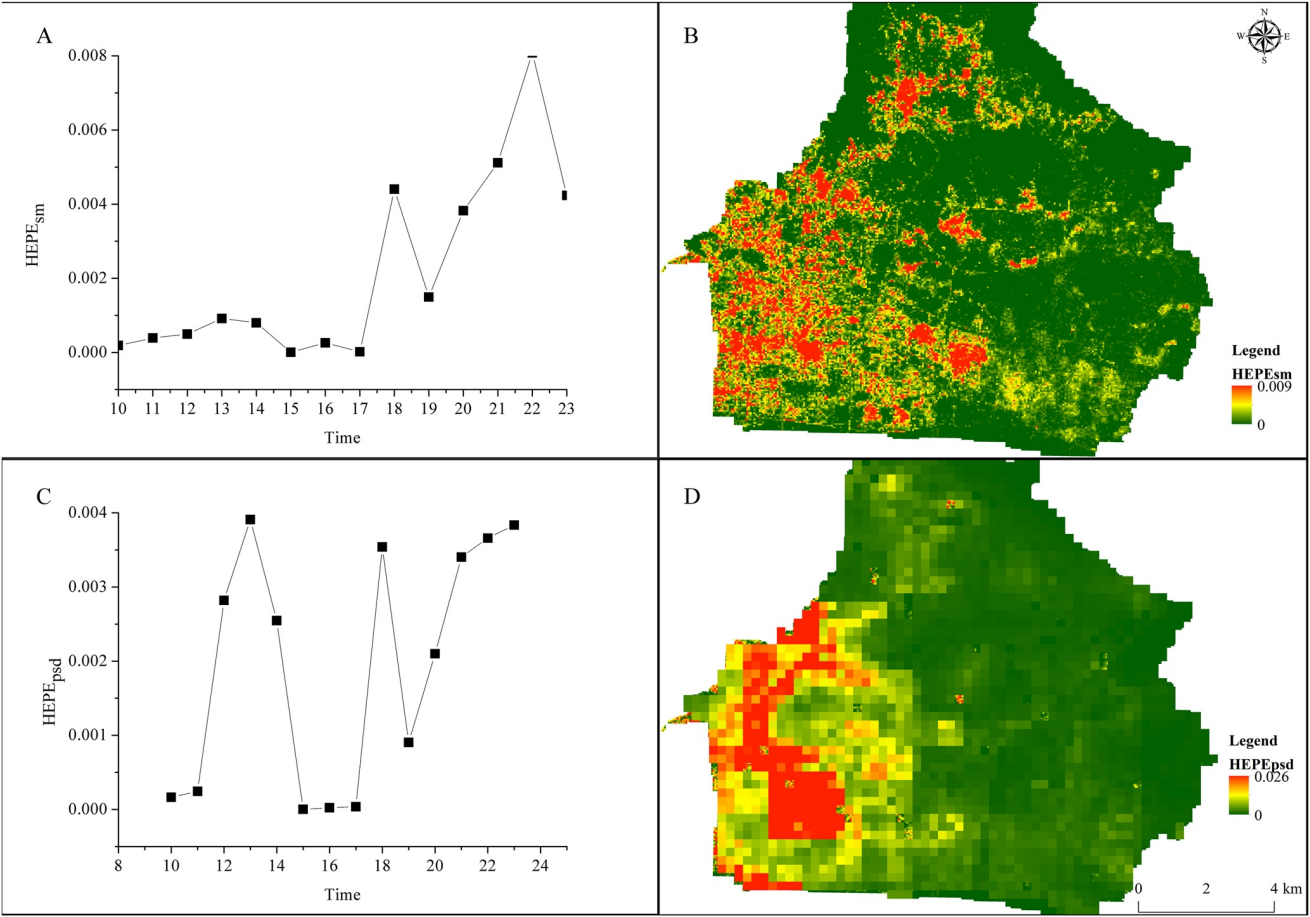
Temporal variations of hourly exceedance PM_2.5_ exposure risk (HEPE)_sm_ and HEPE_psd_ (a and c) and a spatial distribution of daily total HEPE_sm_ and HEPE_psd_ (b and d).

Figure 3 illustrates the dynamics of HEPE_sm_ and HEPE_psd_ during the study period. The mean values of HEPE_sm_ and HEPE_psd_ ranged between 1 × 10^−8^ and 2 × 10^−3^ and 1 × 10^−5^ and 1 × 10^−9^, respectively. The peak values of HEPE_sm_ and HEPE_psd_ ranged between 1 × 10^−8^ and 2 × 10^−3^ and 1 × 10^−5^ and 1 × 10^−9^, respectively. Temporal variations in HEPE_sm_ exhibited three peaks at 13:00, 18:00, and 22:00. The peaks of HEPE_psd_ were observed at the same time as that of HEPE_sm_. Although the peaks occurred simultaneously, differences were observed. The peaks of HEPE_sm_ lagged behind the peaks of HEPE_psd_.

### 4.2 Spatial Patterns of Exceedance PM_2.5_ Exposure Using Different Population Data

**Figure 4:**
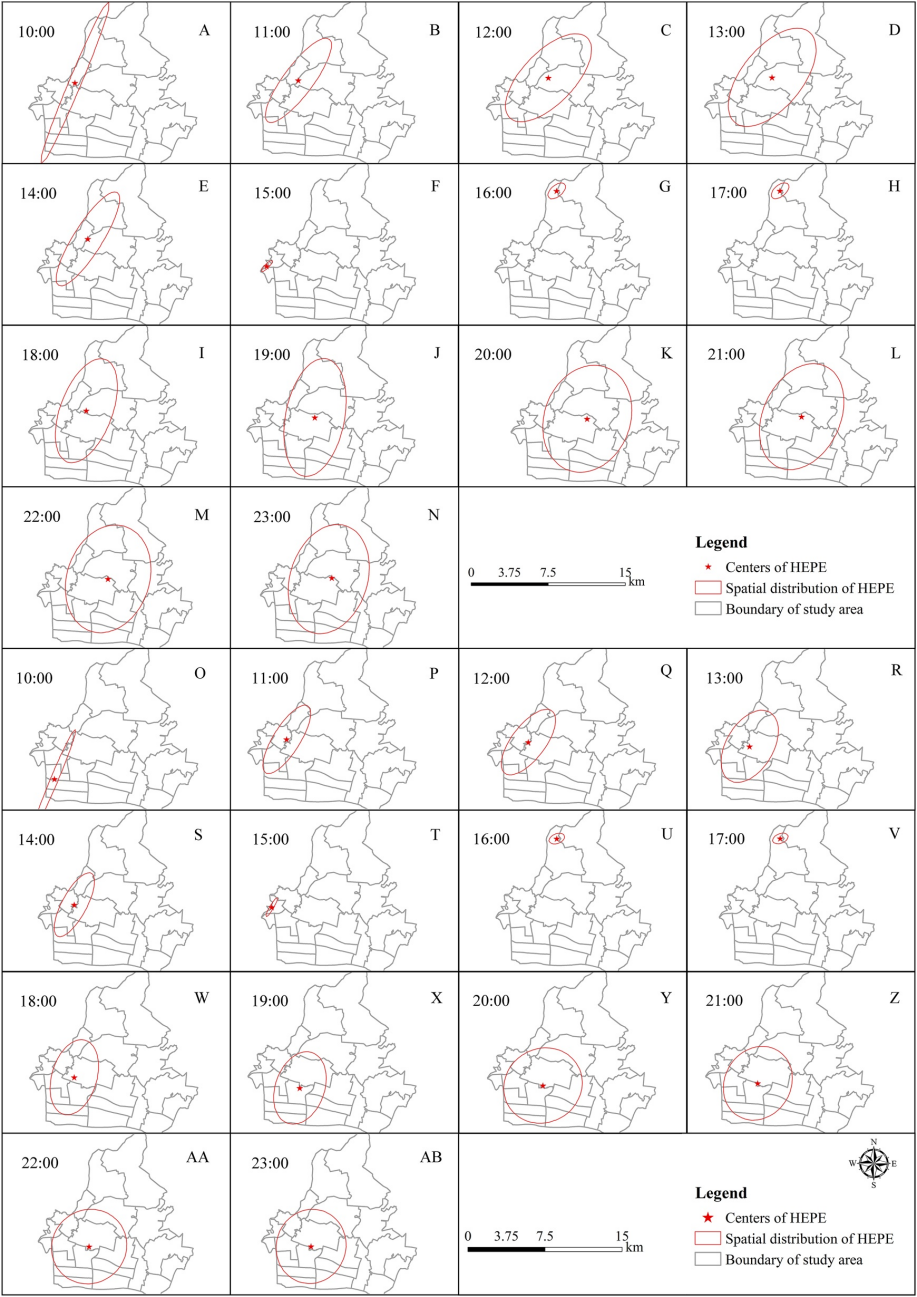
Spatial variations of exceedance PM_2.5_ exposure risk assessed considering social media data and population modeling data. Panels (a–n) show a spatial variation of exceedance PM_2.5_ exposure risk assessed considering the social media data, and panels (o–ab) show a spatial variation of exceedance PM_2.5_ exposure risk assessed considering the population modeling data.

Spatial patterns of hourly HEPE_sm_ and HEPE_psd_ were detected using the SDEM (Figure 4). The spatial centers of HEPE_sm_ were observed at 113.33°E and 23.17°N at 10:00. The spatial center of HEPE_sm_ moved to the northeast of the study area at approximately 113.35°E and 23.18°N. Eventually, the spatial center of HEPE_sm_ was at 113.36°E and 23.16°N. Compared with the spatial center of HEPE_sm_, the spatial center of HEPE_psd_ was located south of the spatial center of HEPE_sm_. It was first observed at 113.31°E and 23.13°N at 10:00. Then, it moved to 113.33°E and 23.16°N. The spatial center of HEPE_psd_ was finally observed at 113.34°E and 23.14°N. The movement of the spatial center of HEPE_sm_ and HEPE_psd_ implied that the public PM_2.5_ exposure risk considering the population modeling data was aggregated to the southwest of the public PM_2.5_ exposure risk considering the social media data.

The major extension direction of HEPE_sm_ was first detected northeast‐southwest at 10:00 and 19:00, then it turned to approximately north‐south. The distance of the long diameter ranged between 1.55 and 10.26 km. The secondary extension direction of HEPE_sm_ was first detected northwest‐southeast at 10:00 and 19:00, then it turned to approximately west‐east. The distance of the short diameter ranged between 0.91 and 7.29 km. The major extension direction and secondary extension direction of HEPE_psd_ were consistent with those of HEPE_sm_. The spatial distribution of HEPE_psd_ was more aggregated with the long diameter ranging between 1.50 and 10.20 km and the short diameter ranging between 0.39 and 7.09 km.

### 4.3 Contribution of Exceedance PM_2.5_ Exposure Risk to the Older Population to the Total

**Figure 5:**
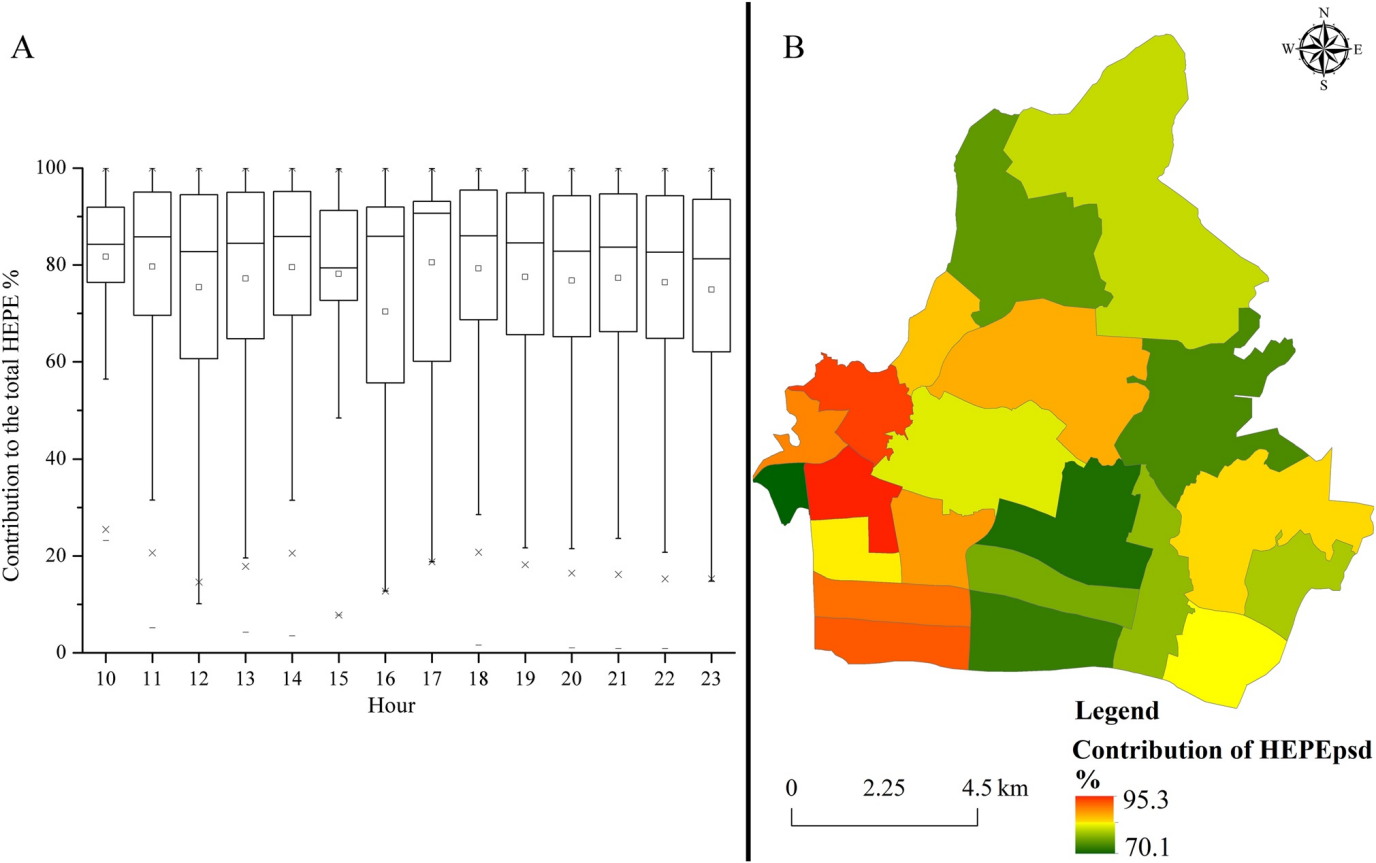
Spatiotemporal variations of contribution of hourly exceedance PM_2.5_ exposure risk (HEPE)_psd_ to the total exceedance PM_2.5_ exposure risk. Panel (a) shows the temporal variations of contribution of HEPS_psd_ to the total exceedance PM_2.5_ exposure risk, and panel (b) indicates the spatial characteristics of contribution of HEPE_psd_ to the total exceedance PM_2.5_ exposure risk.

The contribution of HEPE_psd_ to the total public exceedance PM_2.5_ exposure risk varied spatiotemporally (Figure 5). The average contribution of HEPE_psd_ to the total HEPE ranged from 70.1% to 95.3%. The temporal variations in the contribution of HEPE_psd_ demonstrated two peaks and two troughs. The peaks were observed at 14:00 and 17:00, and the troughs were observed at 12:00 and 16:00. Although the average contribution of HEPE_psd_ was high, the standard deviation was significant. The minimum contribution of HEPE_psd_ ranged from 10.0% to 57.2%. The maximum contribution of HEPS_psd_ was 100%. Four hot spots of contribution were detected, including Xinghua Township, Linhe Township, Xiancun Township, and Liede Township. Cold spots were detected in Shahe Township, Tangxia Township, and Yuancun Township.

## 5 Discussion and Conclusion

Previous studies have documented that the continuing increase in PM_2.5_ poses various health threats, such as premature mortality and excess morbidity, which provide significant information for measuring the harmful effects of ambient air pollution (S. Chen et al., 2020; S. Liu et al., 2020; Lubczyńska et al., 2017; Mortamais et al., 2021; Xue et al., 2019; Yang et al., 2020). The scientific assessment of PM_2.5_ exposure risk is the foundation for these investigations. In our latest investigations, we used social media data to propose a novel indicator named HEPE to provide the significant spatiotemporal characteristics of individual PM_2.5_ exposure risk information. However, due to the nonrepresentative and non‐sample properties, the HEPE for the older adults groups was absent. The total PM_2.5_ exceedance exposure cannot be fully reflected only relying on social media data. Therefore, we proposed to map the total exceedance PM_2.5_ exposure risk by combining the social media data and population modeling data. The theoretical and management implications are as follows.

### 5.1 Theoretical Implications

In previous studies, we first developed the indicator HEPE. Compared with previous indicators, such as daily mean PM_2.5_ or daily peak PM_2.5_, the advantage of HEPE was that it could represent the different exposure intensities and durations within one day, even with the same daily mean concentration. One study conducted in the Pearl River Delta demonstrated significant variations in HEPE, ranging between 50 and 110 units, with a similar daily mean PM_2.5_ that was monitored in four tropical cities. This variation was associated with a maximum mortality rate of 4.43% and a minimum mortality increase of 2.86% in different cities. Therefore, the implementation of the definition of exceedance PM_2.5_ is helpful in quantifying the high spatiotemporal associations between environmental exposure and public health outcomes.

In this study, another theoretical implication was to couple the multisource data on public health‐related topics. Social media data and population modeling data are the newly developed data and the earliest used data in public‐related topics, respectively. Owing to their advantages such as high spatiotemporal resolution and high data accuracy, they have been widely used in urban planning and public health‐related topics (Grasso et al., 2017; Gu et al., 2016; Jung et al., 2019; X. Liu et al., 2017; Martí et al., 2019; Sun, 2020; Tu et al., 2017). However, the social media and population modeling data were used individually. The combination of these two kinds of data was rarely seen due to the differences in the data source, spatial resolution, information representation method, and information expression contents. By developing the HEPE indicator, we quantified the relative individual exposure risks. Because HEPE results describe the relative exposure risk, the normalized results of social media data and population modeling data avoid the mismatches caused by the differences in data source, spatial resolution, information representation method, and information expression contents. Therefore, this study can help researchers gain insights into theory development for the combination of social media data and traditional population data.

Moreover, the limitation and uncertainty caused by different source data should be addressed. Due to the restriction of Tencent user density data, only May 17, 2019 participated in analysis. The methodology in this study was adaptable for different study areas or periods theoretically. However, considering great changes of population mobility patterns on weekdays or weekends, various temporal scales should be considered in the future, such as daily, weekly, monthly, and seasonal, to map the total population exceedance PM_2.5_ exposure risk comprehensively. Therefore, this could avoid two aspects of uncertainty. The first was caused by the heterogeneity of population mobility at different temporal scales. The second was caused by the variation of PM_2.5_, in case of the influence of unpredictable meteorological events. The other uncertainty was caused by the accuracy of aged population group data. The aged population group data were developed based on aged population survey data and residential area data. Aged population survey data were census data, which were relative accurate and precise. However, due to the statistical method and surveyors' professional skills differences, incidental errors were unavoidable. Moreover, the residential area data were obtained from government or remote sensing data; this data was updated with delays resulting in the system errors of spatial distribution of aged population groups. However, the spatial resolution of this data was 100 m, which was relatively a large spatial scale that smoothed the incidental and system errors. This widely used data proved that local and global accuracy of this data can satisfy the population‐related topic investigation.

### 5.2 Management Implications

The life expectancy with improved air quality has been addressed in previous studies (Qi et al., 2020). When the ambient air pollution guideline of PM_2.5_ from the World Health Organization (25 µg/m^3^) was applied, compared with the Chinese National Ambient Air Quality Standard (75 µg/m^3^), 0.14 years of life expectancy is gained. For the older adult groups, increasing PM_2.5_ concentrations are correlated with atherosclerotic plaque systemic oxidative stress and inflammation, which results in a high risk of mortality and morbidity (Brook et al., 2010). Therefore, an exposure risk assessment of the older adult groups and targeting hot spots is crucial for the development of air pollution mitigation strategies.

In this study, the HEPE_psd_ was used to monitor the HEPE for the older adults. We observed a stable spatial distribution area of HEPE_psd_ during the study period. High‐value areas of HEPE_psd_ were constricted within a circle with 10.2 km, which were located in the primitive downtown of Tianhe District. Two conclusions can be drawn: First, the PM_2.5_ exposure risk is related to the mobility characteristics of the older adult groups. Compared with HEPE_sm_, the spatial pattern of HEPE_psd_ shrank. HEPE_sm_ represents the group of young people. This group of people had periodic commuting characteristics. In the morning, the trajectory of HEPE_sm_ begins from the home and ends at the workplace. In the afternoon, the trajectory of HEPE_sm_ begins at the workplace and ends at home. This trajectory forms the cross‐region results of the HEPE_sm_. In contrast, the trip distance of the older population was constricted around their homes. Second, people in the old city center are exposed to higher air pollution risks, especially for the older adults. As urbanization progresses, high‐quality settlement environments, industries, and medical resources aggregate to new urban centers. Due to cheap rent and a low threshold of employment opportunities, the old urban center is experiencing a significant population growth. A large number of older adults reside in this area, generating a large vulnerable population to PM_2.5_. Therefore, the development of a PM_2.5_ exposure risk mitigation strategy for the older adult should consider two key points. First, the development of a PM_2.5_ exposure risk mitigation strategy should focus on small spatial scales. Targeting the older adult groups, green spaces, such as water bodies or plants, should be built within the common mobility distances of the older resident. Second, more effort to reduce the adverse effects of PM_2.5_ on public health should be made targeting the old urban center.

In China, the daily PM_2.5_ concentration threshold was 75 µg/m^3^, which is three times larger than that guided by the WHO. Relative investigations have demonstrated that stricter ambient quality standards have led to more health benefits. In 2016, the Healthy China 2030 blueprint was released. In this blueprint, life expectancy of 79 years by 2030 is one of the most significant goals. To achieve this goal, strict PM_2.5_ guideline standards and the exceedance effects of PM_2.5_ should be conducted. Our study provides new evidence that the exceedance effects of PM_2.5_ are a significant indicator for assessing the PM_2.5_ exposure risk. We suggest that the findings of this study are helpful for policy‐making.

A few limitations of this study must be addressed. Although we mapped the total exceedance PM_2.5_ exposure risk by combining multisource data, HEPE_sm_ and HEPE_psd_ reflected the relative exposure risk rather than the actual exposure risk. TUD represents the relative population density. To map the real total exceedance PM_2.5_ exposure risk, smartphone or social media data that provide counts in real time are urgently needed. Moreover, seasonal variations in PM_2.5_ and the climatic background influence the exposure risk assessment results. In the future, HEPE_sm_ and HEPE_psd_ in different seasons under different climatic conditions should be further investigated.

## Conflict of Interest

All authors declare no financial or personal relationships with other people or organizations that can inappropriately influence their work. There is no professional or other personal interest of any nature or type in any product, service, and/or company that could be construed as influencing the position presented in, or the review of, the manuscript entitled, “Mapping total exceedance PM_2.5_ exposure risk by coupling social media data and population modeling data.”

## Supporting information

Supporting Information S1

Data Set S1

Data Set S2

## Data Availability

The hourly PM_2.5_ monitoring data can be download from China National Environmental Monitoring Center (http://www.cnemc.cn/). TUD data are obtained from Tencent Internet Corporation. Population modeling data are download from Worldpop (https://www.worldpop.org/). Population modeling data are free of charge.
