# Supplementary material for: Mapping Total Exceedance PM2.5 Exposure Risk by Coupling Social Media Data and Population Modeling Data
Source: Geohealth. 2021 Nov 1;5(11):e2021GH000468. doi: 10.1029/2021GH000468 (PMC8576961; doi:10.1029/2021GH000468)
Supplement: Supplementary file 1 — Supporting Information S1 [file GH2-5-e2021GH000468-s003.docx]

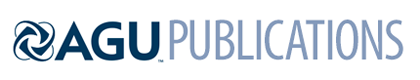


*Geohealth*

Supporting Information for

**[Mapping total exceedance PM2.5 exposure risk by coupling social media data and population modelling data]**

[Zheng Caoa,b, Guanhua Guoa,b, Zhifeng Wua,b*, Shaoying Lia,b, Hui Suna,b, Wenchuan Guana]

[a School of Geographical Sciences, Guangzhou University, Guangzhou 510006, China

b Southern Marine Science and Engineering Guangdong Laboratory (Guangzhou), Guangzhou 511458, China]

**Contents of this file**

Text t1

Figures S1 to S2

**Additional Supporting Information (Files uploaded separately)**

Captions for Datasets S1 to S3

**Introduction**

Text t1

**Data Statement**

The hourly PM2.5 monitoring data can be download from China National Environmental Monitoring Center (<http://www.cnemc.cn/>). TUD data is obtained from Tencent Internet Corporation. Population modelling data is download from Worldpop (<https://www.worldpop.org/>). Hourly PM2.5data and TUD data have been submitted to Geohealth Journal. Population modelling data is free charge.





Figure S1. Population mobility pattern based on TUD in study area.


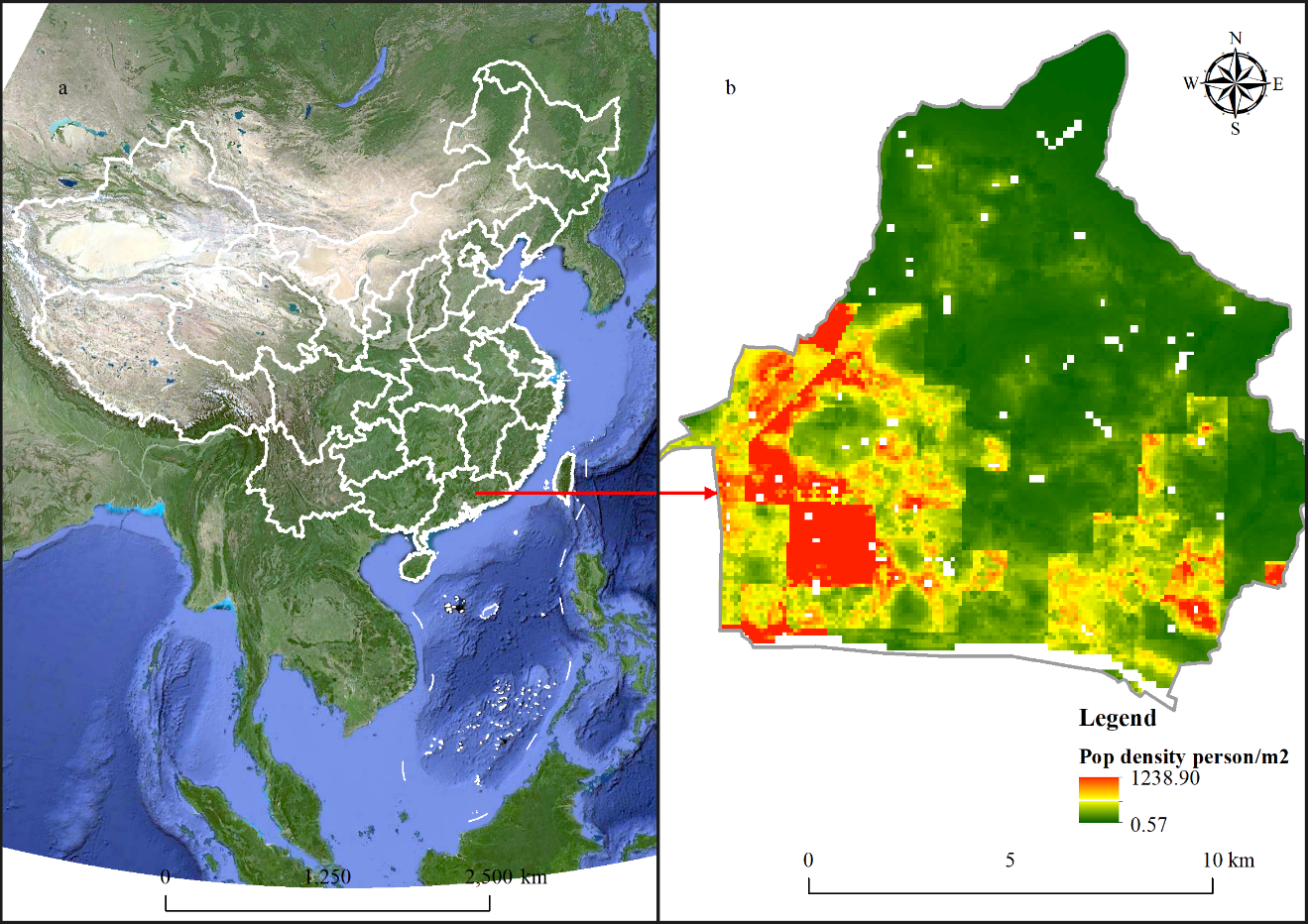


Figure S2. Spatial distribution of the aged population in study area.

Data Set S1. Hourly PM2.5 monitoring data.

Data Set S2. TUD data
